# Socioeconomic characteristics and domestic work as correlates of family satisfaction in Hong Kong mothers of young children

**DOI:** 10.1186/s12889-023-17129-x

**Published:** 2023-11-08

**Authors:** Ester Cerin, Casper J.P. Zhang, Robin R. Mellecker, Wai-kit Ming, Anthony Barnett

**Affiliations:** 1 Mary MacKillop Institute for Health Research, Australian Catholic University, Melbourne, VIC 3000 Australia; 2 School of Public Health, The University of Hong Kong, Pok Fu Lam, Hong Kong SAR; 3 Faculty of Education, The University of Hong Kong, Pok Fu Lam, Hong Kong SAR; 4 Department of Infectious Diseases and Public HealthCity University of Hong Kong, Kowloon, Hong Kong SAR

**Keywords:** Life satisfaction, Psychological wellbeing, Housework, Work-family relation, quality of life

## Abstract

**Background:**

Family life satisfaction is an important contributor to the mental health of mothers with young children, who are particularly vulnerable to various sources of stressors. However, there is a dearth of studies on this topic in this demographic, the determinants of which likely differ across geographical and cultural contexts. We examined indicators of maternal socioeconomic status (SES) and domestic help as correlates of family satisfaction in Hong Kong mothers of young children.

**Methods:**

Mothers (N = 322) of young children (3–5 years old) were recruited from neighbourhoods stratified by SES and population density. They self-completed a survey containing items on socio-demographics, SES characteristics (including household income and maternal education and employment status), maternal family satisfaction and division of domestic work in the household and family. Confounder-adjusted associations of maternal SES indicators and participation in housework and childcare activities by various agents (e.g., mother, spouse, other residents) were estimated. We also estimated the moderating effects of household income on the associations between maternal employment and family satisfaction, and those of maternal employment on the associations between domestic work division and family satisfaction.

**Results:**

Household income and maternal education were positively related to maternal family satisfaction. Mothers in part-time employment had lower family satisfaction than non-working mothers and mothers working full-time. The latter reported higher family satisfaction than non-working mothers only if their household income was below HK$ 15,000. Domestic work performed by non-residents was predictive of higher family satisfaction, while mothers’ housework and child(ren) tutoring were predictive of lower family satisfaction. Only part-time employed mothers benefited from spouse’s assistance with domestic work. The interaction effects of maternal employment status on the associations between the division of child tutoring and family satisfaction were complex.

**Conclusions:**

In Hong Kong, mothers of young children with lower education and household income, who hold a part-time job and participate in housework and child tutoring activities have the lowest levels of family satisfaction and, hence, are at higher risk of mental health problems. Spouses’ and non-resident family members’ participation in domestic work, as well as the establishment of more family-friendly employment practices, may help mitigate this risk.

**Trial registration:**

N/A.

**Supplementary Information:**

The online version contains supplementary material available at 10.1186/s12889-023-17129-x.

## Background

Life satisfaction, of which family life satisfaction is an important component [1], is deemed to contribute to better physical [2, 3] and mental health [4]. Mothers of young, preschool-age children are at increased risk for mental health problems due to being exposed to parenting-related social stressors (e.g., too much time spent on childcare, lack of support from others with childcare) and financial stressors (e.g., lack of resources for child education) [5], which impact their family life satisfaction [6, 7]. It is, therefore, important to identify factors that contribute to better family life satisfaction among mothers of young children.

Indicators of socioeconomic status (SES), encompassing household income and educational attainment, are strong determinants of life satisfaction, including family life satisfaction [8–11]. In general, wealthier and more educated people tend to report higher levels of happiness and family satisfaction compared to their counterparts [10, 12–16]. However, most research in this area has focused on overall rather than family life satisfaction and on broader adult populations. A greater focus on mothers of young children is required to understand how SES affects their family life satisfaction and help identify ways to reduce the risk of mental health problems in this particularly vulnerable group.

Another influential SES indicator related to overall and family life satisfaction is employment status. While employed people are more satisfied with their life than those unemployed [17], the difference in life satisfaction between employed and economically inactive (i.e., not seeking employment) individuals appear to depend on gender. Studies support a positive effect of employment on life and family satisfaction in fathers [18–20], while the evidence about mothers is inconclusive and varies across countries [18–21]. These conflicting findings have been attributed to maternal employment having mixed consequences that vary across cultural contexts. On the one hand, paid employment may increase a mother’s negotiating power in the family [22], enrich her social networks [23] and contribute to the financial costs of raising a family, the latter being a significant stressor for parents [5]. On the other hand, employed mothers may experience higher levels of stress and work-to-family conflicts arising from the diminished time on hand to satisfy childcare and household demands [24] given that, in most society, they are still expected to be the primary caregivers of the household [25–27]. This is a public health concern because work-family conflict is a major determinant of family life satisfaction [28] and psychological well-being [28, 29], especially in working mothers [30].

Work-to-family conflict and its negative effects on maternal life and family satisfaction may be mitigated by a more egalitarian division of domestic work among household members [21, 24, 31] or the outsourcing of domestic work [32–34]. The increase in women’s paid work has been mirrored by a slow gender convergence in the time spent on domestic work [27, 35] and a rise in paid domestic help [36–39]. However, these trends appear to be activity- and country-specific. For example, developed countries have witnessed a sizable reduction in the time mothers spend on housework but an increase in the time they spend on childcare [27]. Increases in childcare time have been also found in fathers, while their participation in housework has remained stable in some countries and increased in others [27]. Paid domestic help has been associated with reductions in time spent on core housework activities, such as cleaning and cooking [40–42], but no reductions in time spent on childcare [42]. The latter phenomenon has been attributed to the widespread belief that intensive parenting – described as ‘child-centred, expert-guided, emotionally absorbing, labour intensive, and financially expensive’ [43] - is optimal for children’s education, and emotional and cognitive development [44]. The differences in temporal trends of maternal time allocated to housework vs. childcare activities suggest that these activities may impact maternal family life satisfaction differently, with housework being more detrimental or less beneficial than childcare to mothers’ levels of satisfaction with family life.

Another understudied factor potentially related to mothers’ wellbeing and family satisfaction is the contribution of other family resident or non-resident members to household labour, which is not surprising as most research on this topic was conducted in Western countries where nuclear and single-parent families are the most prevalent [45]. Extended families (i.e., families with parents, children and grandparents or other adult members) are more common in Asian, Central/South American and sub-Saharan countries [45]. Studies suggest that the presence of grandparents in the household may reduce parents’ time allocated to housework in Asian countries [42, 46–48]. However, it is unclear whether this leads to higher family satisfaction [31].

Because family satisfaction and its correlates vary substantially across countries and cultures, it makes sense to study family satisfaction of mothers of young children in specific geographical and cultural contexts. Here, we focus on Hong Kong Chinese mothers of preschool-aged children and, before delineating the study aims, provide a short overview of the socio-cultural context of Hong Kong underpinning family life.

Family life in Hong Kong is characterised by a mix of Chinese traditionalism typified by collectivism and Western modernism focused on individualism. Attitudes and beliefs about family are based on utilitarianistic familism placing one’s familial interests above those of other individuals, groups and society [55]. Self-interest and economic gains at the family level are the main drivers of the family structure and processes in Hong Kong, and these influence decisions regarding childcare arrangements and maternal employment. Although, as in Western cultures, the nuclear family is the most common familial form, extended family households are more prevalent in Hong Kong than in many Western countries [45, 49]. Furthermore, Hong Kong people still hold some traditional family norms, including the expectation of siblings to perform obligations, the tendency to adhere to traditional gender roles and consider childbearing as the central role of women [50]. Such family structure and norms may create more opportunities for mothers to share household duties with other family members, avert work-to-family conflict given that work is seen to serve a utilitarian function for the benefit of the family [51], but also potentially pose excessive demands on working mothers causing stress and low levels of family satisfaction [21].

Mirroring global trends [52] and owing to a rapid economic growth, the employment rate of Hong Kong mothers with young children has substantially increased in the last two decades [53]. However, it still lags behind many Organisation for Economic Cooperation and Development (OECD) member countries, with Hong Kong reaching a 57% employment rate in 2018 [53] compared to 71% in the OECD [52]. This difference has been attributed to more flexible, family-friendly workplace arrangements and pro-family policies, and a greater and better provision of centre-based childcare services in the OECD than in Hong Kong [53]. Hong Kong mothers who cannot rely on adequate centre-based childcare typically have three options: to give up their job in part or in full; to share childcare with family members (e.g., partner or parents); and/or to hire a domestic helper. The latter option is quite common in Hong Kong with > 350,000 households employing foreign domestic helpers for housework and childcare in 2016 [54]. Yet, even in households with hired domestic helpers, mothers are still responsible for the care and wellbeing of children and older family members [55].

Considering the unique socio-cultural environment of Hong Kong, this study aimed to examine the associations of maternal SES indicators (educational attainment, employment status and household income), having a domestic helper and the division of housework and childcare activities with family satisfaction in Hong Kong mothers of preschool-aged children. As maternal employment may mitigate the financial strains associated with having a child and bring financial gains to the whole family, we also examined whether household income moderated the association between maternal employment and family satisfaction. Furthermore, as the division of housework and childcare is likely to be particularly important for women juggling motherhood and a career, we examined the extent to which maternal employment status moderated the associations between the division of housework and childcare and maternal family satisfaction.

We hypothesised that: (1) mothers with higher SES would report higher levels of family satisfaction; (2) mothers’ employment would be more positively associated with family satisfaction in lower income households; (3) mothers’ engagement in housework but not childcare activities would be negatively associated with family satisfaction; (4) assistance with housework and childcare from others would be positively associated with family satisfaction; and (5) the division of housework and childcare activities would have a stronger positive impact on family satisfaction in working than non-working mothers.

## Methods

We used secondary data from a project on environmental and parental determinants of Hong Kong preschool-age children’s physical activity [56, 57]. Prior to data collection, ethical approval was obtained from the Human Research Ethics Committee for Non-Clinical Faculties of the University of Hong Kong (#EA560310).

### Recruitment and participants

A sample of 411 Chinese-speaking parents or caregivers of preschool-aged children (3–5 years) were recruited in 2011–2014 from 96 neighbourhoods (here, defined as Tertiary Planning Units, TPUs, which are small administrative area units in Hong Kong with Census data) stratified by area-level SES and population density [56, 57]. TPUs with a population density ≤ 9000 and > 9000 residents/km^2^ were, respectively, classified into low-to-medium and medium-to-high density neighbourhoods. The classification of TPUs into low-to-medium vs. medium-to-high SES was based on the median split of monthly domestic income (here, HK$24,500) derived from 2010 to 2011 Census data [63, 64]. Twenty-four TPUs per stratum were randomly selected for participant recruitment in order to maximize the variability in SES and access to services, which are deemed to influence household behaviours and engagement in various activities (e.g., physical activity, playing with children, shopping) [57–59]. Of the 411 parents or caregivers recruited in the study (~ 80% female, mean age 37.2 (SD 5.8) years), 322 were mothers. We examined only data from the latter subgroup because this study focused on mothers of young children. With 322 participants, the study was powered to detect a small effect size corresponding to ~ 2% of explained outcome variance.

Participants were recruited in person at kindergartens and Maternal and Child Health Centres located in the 96 pre-selected TPUs. Eligibility criteria included: being the parent / primary caregiver of at least one Chinese-speaking 3-5-year-old child residing in one of the pre-selected neighbourhoods; and being able to read and write in Chinese. Parents/primary caregivers of children with a disease affecting their physical activity behaviour or cognitive functioning (e.g., physical disability, severe asthma or Down’s syndrome) were excluded from the study. Informed written consent was obtained from each participant prior to data collection. Participants completed a survey containing items on sociodemographic characteristics, family functioning, family life satisfaction, and participation in and distribution of housework and childcare activities. Completed surveys were returned to the research team by reply-paid mail.

### Measures

#### Outcome variable

Maternal family life satisfaction was measured using an 8-item scale employed in the China Housing Survey 1993 [60, 61]. The scale includes items measuring satisfaction with: (1) marital relationship; (2) sexual relationship with spouse; (3) relationship with children; (4) family economic well-being; (5) relationship among family members; (6) relationship with relatives; (7) housework responsibilities; (8) family life in general. The items were scored on a 5-point scale ranging from 1 (*‘very dissatisfied’*) to 5 (*‘very satisfied’*). In this study, the internal consistency (Cronbach’s alpha) of the scale was 0.91. A participant’s score on the family life satisfaction scale was represented by the average rating on the items.

### Explanatory variables

Maternal SES was defined using three indicators, including education level (highest attained), employment status and household income. Education level included the categories *‘Lower secondary or below’*, *‘Higher secondary/diploma’* and *‘Bachelor’s degree or above’*. Employment status was categorised into *‘not currently employed’*, *‘part-time’*, and *‘full-time’*. This variable was used as an explanatory variable as well as a moderator of the associations between indicators of division of household in the family and family life satisfaction. Household income (average monthly) was reported using 10 ordered categories ranging from *‘less than HKD 6,000’* to *‘> HKD 59,999’*. Household income was also treated as a moderator of the associations between maternal employment status and family life satisfaction.

The division of housework in the family was captured by items gauging the presence of a domestic helper (*‘no domestic helper’*, *‘non-resident domestic helper’* and *‘resident domestic helper’*) and items asking the participants to report who performed the following tasks: (1) grocery shopping; (2) cooking meals; (3) laundry; (4) house cleaning; (5) repair work; (6) taking care of parents when they need help; (7) taking care of children when they are sick; (8) tutoring children; (9) playing with children; and (10) shopping for other daily needs. Response options were: *‘respondent’* (mother), *‘spouse’*, *‘other resident’*, *‘parents living elsewhere’*, *‘children living elsewhere’* and *‘other non-residents’*. All tasks except for tutoring children and playing with children were classified as housework. As tutoring children and playing with children are considered childcare activities that benefit a child’s development that mothers’ may particularly value [27], they were examined separately from other household activities. For each mother, we tallied the number of self-reported housework tasks (range: 0 to 8) and recorded whether (yes/no) they reported tutoring or playing with their child(ren). We did the same for tasks performed by their spouse and those performed by other residents excluding resident domestic helpers. Tasks reported to be done by ‘*parents living elsewhere*’, ‘*children living elsewhere*’ and ‘*other non-residents*’ were combined into scores for housework tasks performed by the three categories of non-residents (excluding non-resident domestic helpers) (score range: 0–24), scores for tutoring (score range: 0–2, *NB*: only adult tutors were included) and scores for playing with children (score range: 0–3).

### Covariates

Maternal age (years), number of adults and children in the household, number of hours a preschool-age child spends outside the home (e.g., at childcare), and size of non-resident extended family were identified as covariates. The latter variable was gauged by asking the participants to provide information on all relatives who were not living in their household.

### Data analyses

Descriptive statistics (e.g., frequencies, means and standard deviations) were computed for all variables included in the study, as appropriate. Directed acyclic graphs based on expert knowledge, extant literature and previous findings from the same project [57, 58] were used to identify the minimum set of confounders to be included in models estimating the total effects (associations) of each explanatory variable on maternal family life satisfaction (see Additional file 1). Total effects are represented by the sum of the effects of an explanatory variable on the outcome mediated and unmediated by potential intermediate variables [62]. For example, the total effect of maternal educational attainment on family life satisfaction consists of the sum of the direct effect of maternal educational level on family life satisfaction and the indirect effects of other intermediate variables between educational attainment and family life satisfaction (e.g., household income, having a domestic helper, etc.).

Generalised linear mixed models with random intercept at the administrative unit level were used to estimate the confounder-adjusted associations between explanatory variables and family life satisfaction, and the effects of moderators. Gaussian variance and identity link functions were employed. Diagnostic tests were performed to assess the validity of the models (e.g., examination of violation of assumptions of linearity, homoscedasticity, identification of influential observations). The moderating effects of maternal employment status and household income were examined by including appropriate 2-way interaction terms to the corresponding main effect models. Significant interaction effects were probed by estimating the associations of a specific explanatory variable (i.e., division of domestic work in the family; employment status) with family life satisfaction at specific values of the moderator. All analyses were conducted in STATA 14.2 (StataCorp, 2015, College Station, Texas).

## Results

The characteristics of the sample are reported in Table 1. The number of non-working mothers was similar to that of full-time employed mothers (44.4% vs. 46.0%), while only a small proportion of them reported working part-time (9.6%). More than half of the households did not have a domestic helper. Domestic activities were mainly performed by mothers (89.1% for playing with children and 96.6% for tutoring children). They engaged, on average, in 5.1(± 1.9) types of housework activities. As to their spouses, they performed an average of 2.6(± 2.3) types of housework activities and more than half of them played with children (58.1%) and tutored children (58.1%). A small proportion of mothers reported domestic work performed by other household residents or non-residents, excluding domestic helpers: 18.1% for playing with children; 14.7% for tutoring children; and 12.1% for housework. Mothers’ average family life satisfaction score was 3.7 (± 0.6), positioned between ‘*somewhat satisfied*’ (3 points) and ‘*satisfied*’ (4 points).

**Table 1:** Sample characteristics (N = 322)

Variable	Statistics
***Socio-demographic characteristics***	
Maternal age (years), *M (SD)*	36.1 (4.8)
Single-parent household, % (n)	2.5 (8)
Nuclear-family household, % (n)	66.2 (213)
Living with parents and/or in-laws, % (n)	23.9 (77)
Number of adults in the household, *M (SD)*	2.5 (1.1)
Number of children in the household, *M (SD)*	1.7 (0.7)
Non-resident extended family size, *M (SD)*	3.1 (3.9)
Hours preschool-aged child spends outside home, *M (SD)*	3.3 (0.7)
***Maternal socioeconomic status (explanatory variables), %***	
Education, % (n)	
Lower secondary or below	40.1 (129)
Higher secondary / diploma	22.7 (73)
Bachelor’s degree or above	37.3 (120)
Employment status, % (n)	
Not working	44.4 (143)
Part-time	9.6 (31)
Full-time	46.0 (148)
Household income (month), % (n)	
< HK$ 10,000	7.5 (24)
HK$ 10,000–29,999	43.8 (141)
HK$ 30,000 and over	48.7 (157)
***Division of domestic work (explanatory variables)***	
Domestic helper in the household, % (n)	
No helper	54.5 (176)
Non-resident	23.4 (75)
Resident	22.1 (71)
Domestic activities	
Play with children, % (n)	
Mother	89.1 (287)
Spouse	58.1 (187)
Other residents (excluding domestic helper)	6.2 (20)
Non-residents / extended family	11.9 (38)
Tutor children, % (n)	
Mother	96.6 (311)
Spouse	58.1 (187)
Other resident (excluding domestic helper)	4.4 (14)
Non-residents / extended family	10.3 (33)
Number of housework activities, M (SD)	
Mother	5.1 (1.9)
Spouse	2.6 (2.3)
Other resident (excluding domestic helper)	0.5 (1.6)
Non-residents / extended family (number of activities × number of categories of people assisting with activity^a^)	0.9 (1.7)
***Maternal family life satisfaction (outcome variable)***, M (SD)	3.7 (0.6)

*Note.* HK$ = Hong Kong dollars; *M* = mean; *SD* = standard deviation. Housework activities include grocery shopping, cooking meals, laundry, house cleaning, repair work, shopping for other daily needs, caring for adult family members when they need help and caring for children when they are sick. ^a^ categories of people include: parents living elsewhere, children living elsewhere and other non-residents

*Associations (total effects) of indicators of maternal SES and division of domestic work with family life satisfaction*.

Table 2 reports the confounder-adjusted associations of maternal SES with mothers’ family life satisfaction. Family life satisfaction was higher among mothers with at least higher secondary schooling than those with lower secondary schooling or below. Mothers with higher household income also reported higher family life satisfaction. Part-time employed mothers experienced lower family life satisfaction than non-working mothers (*b*=-0.17; 95% CI -0.34, -0.01; *p* = .045) and mothers in full-time employment (*b*=-0.25; 95% CI -0.41, -0.09; *p* = .002), while there was no significant difference between non-working mothers and those employed full-time (*b* = 0.08; 95% CI -0.05, 0.21; *p* = .242). Household income moderated the association between full-time employment and family life satisfaction, whereby mothers in full-time employment reported higher levels of satisfaction than non-working mothers only if their monthly household income was below HK$ 15,000 (Table 2).

**Fig. 1:**
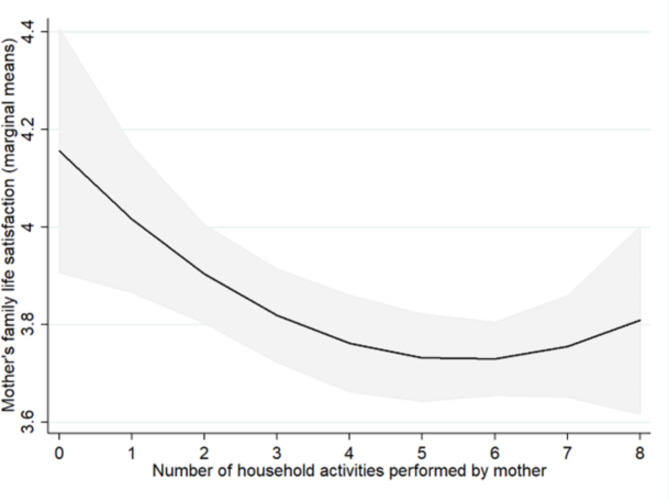
Curvilinear relationship between number of housework activities performed by the mother and maternal family life satisfaction. *Notes*. The solid lines represent point estimates (grey boundaries are 95% confidence intervals)

As to the division of domestic work, activities performed by non-resident extended family members or other non-residents (excluding domestic helpers) were found to be positively associated with maternal family satisfaction (Table 3). Specifically, compared to mothers without such assistance, mothers were more satisfied with their family life if non-residents / members from extended family played with their children, tutored their children or performed housework activities. Mothers who tutored their own children reported lower satisfaction than those who did not (Table 3). Those performing housework activities also reported lower satisfaction. However, this relationship was curvilinear (quadratic) as depicted in Fig. 1. The level of family satisfaction decreased with an increase in the number of housework tasks from 0 to 4, while further increases in the number of housework tasks performed did not result in further decreases in satisfaction. Activities performed by the spouse or other residents in the household were not found to be associated with maternal family life satisfaction.

**Table 2:** Associations (total effects) of maternal socioeconomic status with family life satisfaction

Variable	*b*	95% CI	*p*-value
*Main effect models*			
Education (ref: lower secondary or below)			
Higher secondary / diploma	0.18	0.04, 0.33	0.012
Bachelor’s degree or above	0.21	0.07, 0.35	0.002
Employment status (ref: not working)			
Part-time	-0.17	-0.34, -0.01	0.045
Full-time	0.08	-0.05, 0.21	0.242
Household income^a^	0.05	0.02, 0.08	0.001
***Interaction effect models***			
Employment status (ref: not working) by household income			
Part-time by household income	-0.04	-0.10, 0.02	0.228
Full-time by household income			
Linear term (of household income)	-0.42	-0.68, -0.17	0.001
Quadratic term (of household income)	0.03	0.01, 0.04	0.008
*Interaction probing: effect of full-time employment vs. not working @ levels of household income*			
<HK$ 6,000 / month	1.11	0.62, 1.60	< 0.001
HK$ 6,000–7,999 / month	0.78	0.46, 1.09	< 0.001
HK$ 8,000–9,999 / month	0.50	0.30, 0.70	< 0.001
HK$ 10,000–14,999 / month	0.27	0.11, 0.43	0.001
HK$ 15,000–19,999 / month	0.09	-0.08, 0.27	0.299
HK$ 20,000–24,999 / month	-0.03	-0.22, 0.16	0.746
HK$ 25,000–29,999 / month	-0.10	-0.29, 0.08	0.279
HK$ 30,000–39,999 / month	-0.13	-0.29, 0.04	0.140
HK$ 40,000–59,999 / month	-0.09	-0.24, 0.06	0.217
>HK$ 59,999 / month	-0.01	-0.21, 0.18	0.910

*Note.* Results from generalised linear mixed models with random intercepts at the administrative unit level, Gaussian variance and identity link functions. ref: reference category; ^a^ 10 categories modelled as ordered linear effects; HK$ = Hong Kong dollars. Covariates for each model determined using directed acyclic graphs (see Additional files 1 and 2); *b* = regression coefficient; 95% CI = 95% confidence intervals

**Table 3:** Associations (total effects) of indicators of division of domestic work with maternal family life satisfaction – main effect models

Variable	*b*	95% CI	*p*-value
*Domestic helper in the household (ref: no helper)*			
Non-resident	0.01	-0.11, 0.13	0.882
Resident	0.01	-0.13, 0.15	0.874
*Domestic activities performed by residents* ^*a*^			
Play with children (ref: no)	-0.24	-0.58, 0.11	0.183
Tutor children (ref: no)	-0.36	-0.81, -0.09	0.116
Number of housework activities	-0.04	-0.10, 0.04	0.196
*Domestic activities performed by non-residents / extended family* ^b^			
Play with children^c^ (ref: no)	0.18	0.03, 0.33	0.017
Tutor children^c^ (ref: no)	0.24	0.06, 0.42	0.009
Number of housework activities^d^	0.04	0.01, 0.08	0.009
*Domestic activities performed by spouse*			
Play with children (ref: no)	-0.06	-0.18, 0.07	0.393
Tutor children (ref: no)	-0.07	-0.20, 0.05	0.225
Number of housework activities	-0.01	-0.04, 0.02	0.714
*Domestic activities performed by mother*			
Play with children (ref: no)	-0.16	-0.36, 0.04	0.113
Tutor children (ref: no)	-0.34	-0.55, -0.12	0.002
Number of housework activities^e^			
Linear term	-0.15	-0.29, -0.02	0.024
Quadratic term	0.02	0.00, 0.03	0.049

*Note.* Results from generalised linear mixed models with random intercepts at the administrative unit level, Gaussian variance and identity link functions. ref: reference category; ^a^ excluding domestic helper and spouse; ^b^ excluding domestic helper; ^c^ because the percentage of mothers reporting more than one category of non-residents / extended family playing with children (1.9%) and tutoring children (0.6%) was small, these variables were recoded as 0 (non-residents / extended family not engaging in the specific childcare activity) and 1 (non-residents / extended family engaging in the specific childcare activity); ^d^ number of activities × number of categories of people assisting with activity; ^e^ see Fig. 1. Model covariates were determined using directed acyclic graphs (see Additional files 1 and 2); *b* = regression coefficient; 95% CI = 95% confidence intervals

Table 4 shows maternal employment status-specific associations of indicators of division of domestic work with family life satisfaction for statistically significant interaction effects (*p* < .05). Having household residents (other than the spouse and domestic helper) tutor children was associated with lower family satisfaction in non-working mothers, while such division of childcare did not seem to impact the family satisfaction of mothers employed full-time. For mothers working full-time, having their spouse tutor their children was associated with lower family satisfaction. This association was not found in non-working mothers or mothers working part-time. In mothers working part-time, housework activities performed by the spouse, their spouse’s playing with children and having household residents tutor children were associated with higher satisfaction. In contrast, mothers working part-time had lower levels of satisfaction if they themselves reported playing with children (Table 4). Playing with children undertaken by mothers or their spouses was not associated with maternal family satisfaction in non-working mothers or mothers employed full-time.

**Table 4:** Associations of indicators of division of domestic work with maternal family life satisfaction by maternal employment status

Employment status category – indicator^#^	*b*	95% CI	*p*-value
Mothers not working (n = 143)			
Residents: tutor children	-0.52	-0.68, -0.36	< 0.001
Spouse: play with children	-0.10	-0.29, 0.09	0.305
Spouse: tutor children	0.04	-0.11, 0.19	0.620
Spouse: active housework activities	-0.01	-0.05, 0.04	0.825
Mothers: play with child	-0.15	-0.36, 0.07	0.183
Mothers working part-time (n = 31)			
Residents: tutor children	0.34	0.16, 0.52	< 0.001
Spouse: play with children	0.51	0.22, 0.80	0.001
Spouse: tutor children	0.14	-0.13, 0.42	0.312
Spouse: active housework activities	0.07	0.01, 0.12	0.012
Mothers: play with child	-0.84	-1.30, -0.37	< 0.001
Mothers working full-time (n = 148)			
Residents: tutor children	-0.41	-0.94, 0.12	0.130
Spouse: play with children	-0.12	-0.31, 0.06	0.197
Spouse: tutor children	-0.22	-0.40, -0.03	0.021
Spouse: active housework activities	-0.01	-0.05, 0.02	0.421
Mothers: play with child	-0.08	-0.40, 0.23	0.611

*Note.* Results from generalised linear mixed models with random intercepts at the administrative unit level, Gaussian variance and identity link functions. Model covariates were determined using directed acyclic graphs (see Additional files 1 and 2). Only statistically significant (p < .05) explanatory variable by employment status interaction effects are presented. ref: reference category; *b* = regression coefficient; 95% CI = 95% confidence intervals; ^#^ reference category = no (agent not participating in activity)

## Discussion

This study examined the associations of SES indicators (i.e., household income and maternal educational attainment and employment status) with family life satisfaction in Hong Kong mothers of preschool-aged children. We found lower family life satisfaction among mothers with lower education and lower household income and those working part-time, compared to their counterparts. Mothers in full-time employment had higher family life satisfaction than non-working mothers only if their household income was low (< HK$ 15,000). We also examined the associations of maternal family life satisfaction with the division of domestic work and the related moderating role of maternal employment status. Domestic activities performed by non-residents were associated with mothers reporting higher family satisfaction, while mothers tutoring children and doing housework had lower family satisfaction, irrespective of their own employment status. In contrast, maternal employment status determined the relationships between maternal satisfaction with family life and specific domestic activities performed by the mother, her spouse or other family members in the household.

### Maternal SES and family life satisfaction

The positive relationships between two indicators of maternal SES (household income and educational attainment) and family life satisfaction found in the present study are consistent with earlier research. Positive associations between income and life satisfaction have been reported at both country [63] and household levels [8, 14, 64]. In general, people tend to be more satisfied with their life when they have access to disposable resources in addition to those covering basic needs [65]. Household income becomes particularly significant when raising a young family because, as noted earlier, having children may lead to financial stress [5]. The widespread endorsement of utilitarian familism in Hong Kong, focusing on familial economic gains [66] and raising successful and socially competitive children [67], may further amplify the importance of household income for family satisfaction in Hong Kong mothers [68].

Regarding the positive association between maternal educational attainment and family satisfaction found in our study, we need to note that it was observed only in regression models unadjusted for household income (i.e., total effect model in Table 2 and other models listed in Additional files 1 and 2, available on request). In line with these findings, higher income has been proposed as one of the key mechanisms through which higher education leads to higher levels of life satisfaction [69, 70]. A study conducted in four East Asian regions found household income to be particularly important for life satisfaction in China [69]. Another contributing mechanism underpinning the aforementioned association could be mothers’ perception of being more prepared for various aspects of life, including childrearing, as a result of formal education [15].

In terms of maternal employment status, we found that mothers working part-time were less satisfied with their family life compared to their counterparts, and mothers in full-time employment had higher family satisfaction than non-working mothers only if they were from a lower-income household (within the 37th percentile in 2016 [71]). In a similar fashion, a study conducted in four East Asian regions reported that part-time employed women in China had lower marital satisfaction than those who were in full-time employment, but no significant difference in satisfaction was found between full-time employed and non-working wives [21]. Part-time employment may cause lower family satisfaction because it usually entails poorly paid, unskilled jobs with limited prospect for promotion [72] and with non-standard work schedules that generate work-to-family conflict [50]. The Hong Kong Government has been promoting family-friendly employment practices, including flexible working hours and reasonable part-time jobs [53]. However, unlike many OECD’s countries, the implementation of such practices is left to the employer’s discretion [53]. Moreover, the relatively small percentages of mothers working part-time in this study (Hong Kong) and in mainland China [21] suggest that full-time rather than part-time employment may be the accepted norm for Hong Kong Chinese mothers who choose or need to work. Under this assumption, it is possible that some mothers with part-time jobs would have preferred full-time employment but were unable to secure it or would have preferred being a full-time homemaker but could not afford it, which would have negatively affected their family satisfaction.

Mirroring the distribution of labour force participation rates of Hong Kong mothers with children aged < 15 years [53], over 44% of mothers in our study were not employed. The fact that non-working mothers showed lower family satisfaction than those working full-time only if they lived in lower-income households suggests that, for mothers of disadvantaged households, full-time employment is a pure necessity to meet the financial demands of raising a family and being able to do so has, understandably, a major positive impact on their family satisfaction. Mothers living in less disadvantaged households have more freedom to choose between having a career or devoting all their time to their family and children, which, according to preference theory [73], should make non-working and full-time employed mothers equally satisfied with their family life [18, 74], as this study suggests. In addition, the similar levels of family satisfaction found in full-time working and non-working mothers from less disadvantaged Hong Kong households could be due to maternal employment being a family decision [37], which is in accordance with utilitarian familism [66].

Yet, it should be noted that the average level of family satisfaction in the examined sample fell around being “somewhat satisfied” and “satisfied”, indicating the presence of challenges in both non-working and working mothers. Being a homemaker may be a personal and/or family decision but also reflect the lack of jobs suited to mothers of young children and/or the lack of appropriate and affordable childcare. For example, in 2018, 10% of 30-59-year-old Hong Kong homemakers expressed their willingness to work if employment was located near their home and employment with adequate renumeration and flexible working hours was on offer [53]. Childcare services in Hong Kong have been found to be inadequate in terms of supply, affordability, location and hours of service, making it difficult for mothers of young children to enter the workforce [53].

### Domestic work and family life satisfaction

We expected that maternal participation in housework activities would negatively impact their family satisfaction but participation in activities benefiting their child’s development would not. This hypothesis was generally supported by the data, with exception of child tutoring and playing with children in mothers working part-time, as detailed below. The J-shaped negative association between the number of different types of housework performed by mothers and their family satisfaction suggests that the most negative effects arose from increasing the number of activities from 0 to 4. Yet, these effects amounted to a difference of only 0.3 points on the family satisfaction scale (Fig. 1).

Our hypothesis that assistance with housework and childcare from others, including a domestic helper, would be positively associated with maternal family satisfaction was only in part supported and, generally, more applicable to mothers in part-time employment. Non-working and full-time employed mothers did not appear to benefit from spouses’ and other residents’ assistance. Also, mothers, in general, did not show higher family satisfaction if they had a domestic helper, but they benefited from having non-resident family members help with any domestic work. The lack of evidence of a positive impact of paid domestic helpers on maternal family satisfaction might be due to the fact that they do not reduce the time mothers spent on domestic work because the reduction in housework resulting from having them is offset by an increase in time allocated to developmental childcare activities and managing the helper(s) [42].

In general, our findings are in contrast to the positive effects of spouses’ participation in domestic work on maternal life [24] and family satisfaction [21] reported by multi-country studies, but in line with data from mainland China [21]. Domestic work sharing between husbands and wives appears to be more common in China than Western [24] and other East Asian countries [21]. In our study, over 85% of mothers reported some domestic work done by their spouse and 42.6% reported spouse’s engagement in all three examined domestic activities. In a recent Chinese study, spouse’s participation rate was 71.6% [75] and, of four East Asian regions, China had the smallest domestic work time gap between men and women [35] and the highest spouse’s participation rate in housework (cooking, cleaning the house and doing the laundry) [47]. If domestic work sharing between husband and wife, as well as other adult resident family members, is prevalent and considered a cultural norm, it is not surprising that they were, generally, unrelated to maternal family satisfaction. The positive effects of non-resident family members’ contributions to domestic work on maternal family satisfaction could be attributed to mothers and their spouses having more free time for themselves [44] or having more opportunities to bond with their extended family.

In general, we found that assistance with housework and childcare activities could be the most beneficial to part-time working mothers. The family- and work-related expectations of Hong Kong mothers in part-time employment may exceed their physical and mental capacity and, thus, cause dissatisfaction with family life. Part-time jobs in Hong Kong are usually lower paid and insufficiently flexible to meet the need of mothers with young children. In addition, part-time employment may be perceived by family members as providing sufficient time to mothers to accomplish most domestic work. For these reasons, our study suggests that, in the context of Hong Kong, part-time working mothers of young children are likely to experience high levels of work-to-family and/or family-to-work conflicts. As this group of mothers are less likely to be able to hire a domestic helper (NB: in this study, over 73% of part-time employed mothers did not have a domestic helper) and afford professional tutoring for their children, sharing domestic work with other resident members may possibly counteract the lower levels of family life satisfaction associated with part-time employment.

Among the findings related to the effects of assistance with childcare activities on family satisfaction, it is interesting to note that mothers in full-time employment were less satisfied if their spouse tutored their children. Although this finding may be due to uncontrolled confounders, such as spouse’s work status, it could be argued that mothers in full-time employment are typically more educated and, hence, likely to be more confident in their opinions and disagree with their spouse on child development matters. Such disagreements may reduce their family satisfaction [76]. It is also possible that full-time employed mothers would prefer for their children high-quality professional tutoring to their spouse’s tutoring. In support of this conjecture, the higher likelihood of Hong Kong mothers of young children to be in full-time employment in families of high-income husbands has been attributed to them having high expectations for their children’s academic success and the belief that this can be achieved via costly professional education and extracurricular activities for their children [37].

Among non-working mothers, having household family members other than their spouse tutor their child(ren) was predictive of lower family satisfaction. At the same time, their tutoring children was associated with lower family satisfaction, while having children tutored by non-residents, which in 91% of the cases were grandparents, predicted higher satisfaction. Albeit the latter two effects were also observed in working mothers. The way the division of child tutoring impacts maternal family satisfaction depends on maternal beliefs about the utility of tutoring activities for their children’s development and future academic success, confidence in their competence to successfully tutor their children [77] and time pressure associated with having multiple roles (housework, childrearing and paid work) [75]. In general, mothers, irrespective of their employment status, may not feel they can provide the best education to their child(ren), either because they do not feel competent or have insufficient time to devote to such activities. These feelings may be exacerbated by the ideology of intensive parenting [43], which is common in Hong Kong [42]. Having non-residents parents or in-laws who can mind and tutor the child(ren) outside the home can alleviate maternal feelings of incompetence and time pressure, and also provide some private time for the mother and their spouse, which is especially important for mothers’ wellbeing [44]. Yet, this does not explain the negative effect of child tutoring by resident family members on non-working mothers’ family satisfaction. In this case, dissatisfaction may arise from disagreements related to the way children are being educated. Non-working mothers may be more aware than working mothers of the practices adopted by their resident parents or in-laws given that they live in the same household and spend more time together.

### Strengths and limitations

This study has several strengths. We examined correlates of family life satisfaction, an important component of life satisfaction, in mothers of young children who are a vulnerable, yet overlooked, group. The findings of this and similar studies can inform family policies and practice to increase family satisfaction in this demographic and boost the wellbeing of families as a whole. Unlike earlier studies, we examined the independent contributions of different types of domestic work performed by key agents (e.g., mothers, spouses and other family members) to maternal family satisfaction. Our investigation of the moderating roles of household income and maternal employment status on the associations of maternal family satisfaction with maternal employment status and the division of domestic work, respectively, helped elucidate the interaction between work and family life in Hong Kong mothers of young children. These findings are potentially generalisable to populations with similar demographic and cultural characteristics (e.g., mothers in mainland China and other East Asian regions). This study is not void of limitations. These include the cross-sectional nature of the study that precludes causal inference; the use of non-probability sampling that is susceptible to sampling bias; the lack of information on the time allocated to domestic work by the various agents; the inability to differentiate between unemployed mothers seeking work and non-working mothers; the lack of information on the type of domestic work performed by the paid domestic helper; the lack of detailed information on maternal occupation that could provide a more comprehensive understanding of the role of work-family conflict in influencing family satisfaction among working mothers; and the relatively small number of participants (mothers) in part-time employment raising concerns about the robustness of the findings pertaining to the moderating role of mothers’ employment status on the associations between division of housework and mothers’ family life satisfaction.

## Conclusions

Lower family life satisfaction found in less advantaged Hong Kong mothers of young children (e.g., those with lower education, lower household income and those in part-time employment) requires that particular attention be given to the subjective wellbeing of these mothers and their families. Housework chores and child tutoring activities can also compromise mothers’ satisfaction with family life. These negative effects may be counteracted by family members assisting with housework or childcare so that mothers can find some private time to devote to themselves and their spouse. Mothers with part-time jobs, who may experience more time pressure and demands at work as well as at home, may benefit the most from their spouse’s assistance with housework chores and playing with children, and from the assistance of other resident family members with child tutoring. This study also highlights some findings unique to Hong Kong and culturally and economically similar locations in East Asia. The employment rate among mothers of young children was lower than that observed in many OECD’s countries, a phenomenon that has been attributed to the lack of family-friendly employment practices and the lack of affordable and satisfactory centre-based childcare services in Hong Kong [53]. Government efforts aimed at solving these barriers to maternal employment may significantly improve family satisfaction in mothers of young children and the wellbeing of the whole family.

### Electronic supplementary material

Below is the link to the electronic supplementary material.

Supplementary Material 1

Supplementary Material 2

## Data Availability

The datasets analysed during the current study are not publicly available due to lack of participant consent to share data outside the team of investigators but are available from the corresponding author on reasonable request.

## References

[1] Diener E, Diener M (1995). Cross-cultural correlates of life satisfaction and self-esteem. J Pers Soc Psychol.

[2] Boehm JK, Peterson C, Kivimaki M, Kubzansky LD (2011). Heart health when life is satisfying: evidence from the Whitehall II cohort study. Eur Heart J.

[3] Martín-María N, Miret M, Caballero FF, Rico-Uribe LA, Steptoe A, Chatterji S (2017). The impact of subjective well-being on mortality: a meta-analysis of longitudinal studies in the general population. Psychosom Med.

[4] Fergusson DM, McLeod GF, Horwood LJ, Swain NR, Chapple S, Poulton R (2015). Life satisfaction and mental health problems (18 to 35 years). Psychol Med.

[5] Mistry R, Stevens GD, Sareen H, De Vogli R, Halfon N (2007). Parenting-related stressors and self-reported mental health of mothers with young children. Am J Public Health.

[6] Milovanska-Farrington S, Farrington S (2022). Happiness, domains of life satisfaction, perceptions, and valuation differences across genders. Acta Psychol (Amst).

[7] Dong S, Dong Q, Chen H (2022). Mothers’ parenting stress, depression, marital conflict, and marital satisfaction: the moderating effect of fathers’ empathy tendency. J Affect Disord.

[8] Moss E, Willoughby BJ (2018). Associations between beliefs about marriage and life satisfaction: the moderating role of relationship status and gender. J Fam Stud.

[9] Orellana L, Schnettler B, Adasme-Berríos C, Lobos G, Miranda-Zapata E, Lapo M (2022). Family profiles based on family life satisfaction in dual-earner households with adolescent children in Chile. Fam Process.

[10] Tynes SR (1990). Educational heterogamy and marital satisfaction between spouses. Soc Sci Res.

[11] Aysan MF, Aysan U, Bilgin MH, Danis H, Demir E, Can U (2017). The effect of employment status on life satisfaction in Europe. Empirical studies on Economics of Innovation, Public Economics and Management.

[12] Ball R, Chernova K (2008). Absolute income, relative income, and happiness. Soc Indic Res.

[13] Myers DG (2000). The funds, friends, and faith of happy people. Am Psychol.

[14] Botha F, Booysen F, Wouters E (2018). Satisfaction with family life in South Africa: the role of socioeconomic status. J Happiness Stud.

[15] Salinas-Jiménez MM, Artés J, Salinas-Jiménez J (2011). Education as a positional good: a life satisfaction approach. Soc Indic Res.

[16] Melin R, Fugl-Meyer KS, Fugl-Meyer AR (2003). Life satisfaction in 18- to 64-year-old swedes: in relation to education, employment situation, health and physical activity. J Rehabil Med.

[17] Schoon I, Hansson L, Salmela-Aro K (2005). Combining work and family life: life satisfaction among married and divorced men and women in Estonia, Finland, and the UK. Eur Psychol.

[18] Schröder M (2020). Men lose life satisfaction with fewer hours in employment: mothers do not profit from longer employment—evidence from eight panels. Soc Indic Res.

[19] Pollmann-Schult M (2014). Parenthood and life satisfaction: why don’t children make people happy?. J Marriage Fam.

[20] Hori M, Kamo Y (2018). Gender differences in happiness: the effects of marriage, social roles, and social support in East Asia. Appl Res Qual Life.

[21] Hori M (2017). Full-time employment and marital satisfaction among women in east Asian societies. Comp Sociol.

[22] Coltrane S (2000). Research on household labor: modeling and measuring the social embeddedness of routine family work. J Marriage Fam.

[23] Ross CE, Mirowsky J, Goldsteen K (1990). The impact of the family on health: the decade in review. J Marriage Fam.

[24] Treas J, van der Lippe T, ChloeTai TO (2011). The happy homemaker? Married women’s well-being in cross-national perspective. Soc Forces.

[25] Cotter D, Hermsen JM, Vanneman R (2011). The end of the gender revolution? Gender role attitudes from 1977 to 2008. Am J Sociol.

[26] Lee YJ, Tsai M-C, Chen W-c (2017). Multiple dimensions of gender-role attitudes: diverse patterns among four East-Asian societies. Family, Work and Wellbeing in Asia.

[27] Pailhé A, Solaz A, Stanfors M (2021). The great convergence: gender and unpaid work in Europe and the United States. Popul Dev Rev.

[28] Amstad FT, Meier LL, Fasel U, Elfering A, Semmer NK (2011). A meta-analysis of work-family conflict and various outcomes with a special emphasis on cross-domain versus matching-domain relations. J Occup Health Psychol.

[29] Spector PE, Cooper CL, Poelmans S, Allen TD, O’Driscoll M, Sanchez JI (2004). A cross-national comparative study of work-family stressors, working hours, and well-being: China and Latin America versus the anglo world. Pers Psychol.

[30] Walsh E, Murphy A (2021). Life satisfaction amongst working parents: examining the case of mothers and fathers in Ireland. Int J Soc Econ.

[31] Forste R, Fox K (2012). Household labor, gender roles, and family satisfaction: a cross-national comparison. J Comp Fam Stud.

[32] Anderson B (2001). Just another job? Paying for domestic work. Gend Dev.

[33] Chan AH-n (2005). Live-in foreign domestic workers and their impact on Hong Kong’s Middle Class families. J Fam Econ Issues.

[34] Cheung AK-L (2014). Hiring domestic help and family well-being in Hong Kong: a propensity score matching analysis. J Comp Fam Stud.

[35] Kan M-Y, Zhou M, Kolpashnikova K, Hertog E, Yoda S, Jun J (2022). Revisiting the gender revolution: time on paid work, domestic work, and total work in east Asian and western societies 1985–2016. Gend Soc.

[36] de Ruijter E (2004). Trends in the outsourcing of domestic work and childcare in the Netherlands: compositional or behavioral change?. Acta Sociol.

[37] Tong Y, Chiu SW-k (2017). Women’s labor force participation in Hong Kong: 1991–2011. Chin Sociol Rev.

[38] Kornrich S, Roberts A (2018). Household income, women’s earnings, and spending on household services, 1980–2010. J Marriage Fam.

[39] Constable N (2007). Maid to order in Hong Kong.

[40] Craig L, Perales F, Vidal S, Baxter J (2016). Domestic outsourcing, housework time, and subjective time pressure: new insights from longitudinal data. J Marriage Fam.

[41] Van Der Lippe T, Tijdens K, De Ruijter E (2004). Outsourcing of domestic tasks and time-saving effects. J Fam Issues.

[42] Cheung AK-L, Lui L (2022). Does live-in domestic help reduce unpaid household labor? The paradox of intensive parenting and domestic outsourcing. Curr Sociol.

[43] Hays S (1996). The cultural contradictions of motherhood.

[44] Faircloth C, Carter J, Arocha L (2020). Utterly heart-breaking and devastating’: couple relationships and intensive parenting culture in a time of ‘cold intimacies. Romantic relationships in a time of ‘Cold intimacies’.

[45] Scott ME, Wilcox WB, Ryberg R, DeRose L. World family map 2015: mapping family change and child well-being outcomes. New York, NY; 2015.

[46] Lu ZZ, Maume DJ, Bellas ML (2000). Chinese husbands’ participation in household labor. J Comp Fam Stud.

[47] Iwai N, Tsai M-C, Chen W-c (2017). Division of housework in Japan, South Korea, China and Taiwan. Family, Work and Wellbeing in Asia.

[48] Tsuya NO, Bumpass LL, Choe MK (2000). Gender, employment, and housework in Japan, South Korea, and the United States. Rev Soc Policy.

[49] Census, Department S, Table. E2016D: 2016 Population By-census - Main Tables (Household) [updated 27 Feb 2017. Available from: https://www.censtatd.gov.hk/en/EIndexbySubject.html?scode=459&pcode=D5211604#section3.

[50] Oishi AS, Chan RKH, Wang LL-R, Kim J-H (2015). Do part-time jobs mitigate workers’ work–family conflict and enhance wellbeing? New evidence from four East-Asian societies. Soc Indic Res.

[51] Yang N (2005). Individualism-collectivism and work-family interfaces: a Sino-U.S. comparison. Work and family: an international research perspective.

[52] Organisation for Economic Co-operation and Development. Walking the tightrope: Background brief onparents’ work-life balance across the stages of childhood 2016 [Available from: https://www.oecd.org/social/family/Background-brief-parents-work-life-balance-stages-childhood.pdf.

[53] Legislative Council Secretariat. Opportunities and challenges facing maternal workforce in Hong Kong Hong Kong SAR: Legislative Council Secretariat; 2019 [Available from: https://www.info.gov.hk/gia/general/201907/16/P2019071600332.htm.

[54] Immigration Department HKSAR, Statistics on the number of Foreign Domestic Helpers in Hong Kong. 2022 [updated 1 Mar 2022. Available from: https://data.gov.hk/en-data/dataset/hk-immd-set4-statistics-fdh/resource/b983aa1d-2617-4051-9ec1-dc5ca281b117.

[55] Cheung FM, Tang CSK, Brown CM, Gielen UP, Gibbons JL, Kuriansky J (2017). Women’s lives in Contemporary Chinese societies. Women’s evolving lives: global and psychosocial perspectives.

[56] Cerin E, Suen YN, Barnett A, Huang WYJ, Mellecker RR (2017). Validity of a scale of neighbourhood informal social control relevant to pre-schoolers’ physical activity: a cross-sectional study. SSM Popul Health.

[57] Suen YN, Cerin E, Barnett A, Huang WYJ, Mellecker RR (2019). Associations of socio-demographic, family, and neighborhood factors with physical activity-related parenting practices among Hong Kong preschoolers’ parents. Matern Child Health J.

[58] Carver A, Akram M, Barnett A, Mellecker R, Cerin E. Socioeconomic status and physical activity among mothers of young children in an Asian city: the mediating role of household activities and domestic help. Int J Environ Res Public Health. 2020;17(7). 10.3390/ijerph17072498 PMC7178123 32268519

[59] Cerin E, Conway TL, Adams MA, Barnett A, Cain KL, Owen N (2018). Objectively-assessed neighbourhood destination accessibility and physical activity in adults from 10 countries: an analysis of moderators and perceptions as mediators. Soc Sci Med.

[60] Logan JR, Bian Y, China Housing, Survey. 1993. Inter-university Consortium for Political and Social Research [distributor]; 2000.

[61] Ji J, Norling AM (2004). Sexual satisfaction of married urban Chinese. J Dev Soc.

[62] Cerin E (2010). Ways of unraveling how and why physical activity influences mental health through statistical mediation analyses. Ment Health Phys Act.

[63] Deaton A (2008). Income, health, and well-being around the world: evidence from the Gallup World Poll. J Econ Perspect.

[64] Yuan H (2016). Structural social capital, household income and life satisfaction: the evidence from Beijing, Shanghai and Guangdong-province, China. J Happiness Stud.

[65] Diener E, Biswas-Diener R (2002). Will money increase subjective well-being? A literature review and guide to needed research. Soc Indic Res.

[66] Lau SK (1982). Society and politics in Hong Kong.

[67] Anderson T, Kohler HP (2013). Education Fever and the east Asian fertility puzzle. Asian Popul Stud.

[68] Gao X, Lee K, Permpoonputtana K, Plitponkarnpim A. Earning too little and worrying too much: the role of income and financial worries on parents’ well-being in Hong Kong and Bangkok. J Fam Econ Issues. 2022. 10.1007/s10834-022-09863-y PMC9473456 36124141

[69] Chen WC (2012). How education enhances happiness: comparison of mediating factors in four east Asian countries. Soc Indic Res.

[70] Powdthavee N, Lekfuangfu WN, Wooden M (2015). What’s the good of education on our overall quality of life? A simultaneous equation model of education and life satisfaction for Australia. J Behav Exp Econ.

[71] Census, Department S, Hong Kong SAR. 2016 Population By-census. thematic report: Household income distribution in Hong Kong [updated Jun 2017. Available from: https://www.bycensus2016.gov.hk/data/16bc-household-income.pdf.

[72] Tilly C (1996). Half a job: bad and good part-time jobs in a changing labor market.

[73] Hakim C (2003). A new approach to explaining fertility patterns: preference theory. Popul Dev Rev.

[74] Revilock CK. Comparison of mental health status of employed and nonemployed mothers with preschool children. Occup Health Nurs. 1982;30(4):11 – 5, 55. 10.1177/216507998203000401 6917962

[75] Yu X, Liu S. Female labor force status and couple’s marital satisfaction: A Chinese analysis. Front Psychol. 2021;12. 10.3389/fpsyg.2021.691460 PMC8343394 34367016

[76] Tavakol Z, Nasrabadi AN, Moghadam ZB, Salehiniya H (2019). The presence of the child, the opportunity or a threat to marital satisfaction: a qualitative study. J Educ Health Promot.

[77] Yamamoto Y, Holloway SD, Suzuki S (2016). Parental engagement in children’s education: motivating factors in Japan and the U.S. Sch Comm J.

