# Supplementary material for: Socioeconomic characteristics and domestic work as correlates of family satisfaction in Hong Kong mothers of young children
Source: BMC Public Health. 2023 Nov 8;23:2196. doi: 10.1186/s12889-023-17129-x (PMC10631154; doi:10.1186/s12889-023-17129-x)

Additional file 1

Figure S1. Example of a directed acyclic graph used to identify the minimal set of confounders to be included in the model of total effect of housework activities performed by mothers on their family life satisfaction

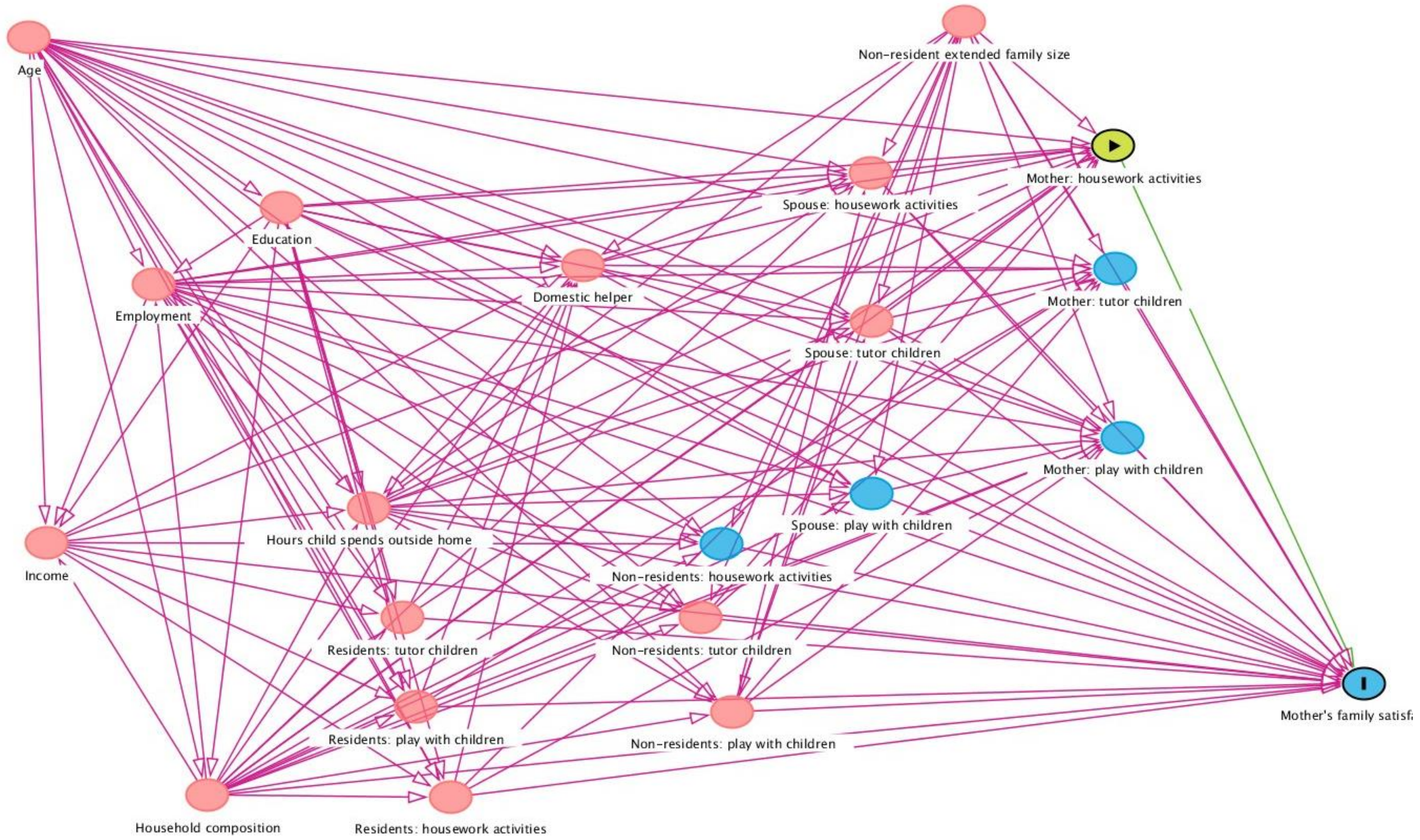

Supplement: Supplementary file 1 — Supplementary Material 1 [file 12889_2023_17129_MOESM1_ESM.pdf]
