# Supplementary material for: Socioeconomic characteristics and domestic work as correlates of family satisfaction in Hong Kong mothers of young children
Source: BMC Public Health. 2023 Nov 8;23:2196. doi: 10.1186/s12889-023-17129-x (PMC10631154; doi:10.1186/s12889-023-17129-x)
Supplement: Supplementary file 2 — Supplementary Material 2 [file 12889_2023_17129_MOESM2_ESM.pdf]

Additional file 2

Table S1. Covariates included in regression models of total effects of indicators of maternal socioeconomic status and division of domestic work on maternal family life satisfaction

Variable	Covariates*
Mother's socioeconomic status	
Education	Age
Employment status	Age, household composition, education
Household income	Age, household composition, employment status, education
Household income by Employment status interaction	As above
Indicators of division of domestic work	
Domestic helper in the household	Age, household composition, employment status, education, household income, non-resident extended family size, residents: play with children, residents: tutor children; residents: housework activities
Employment status by Domestic helper interaction	As above
Domestic activities performed by residents (excluding domestic helper)	
Play with children	Age, household composition, employment status, education, household income
Tutor children	As above
Number of housework activities	As above
Employment status by Domestic activities performed by residents interaction	As above
Domestic activities performed by non-residents / extended family (excluding domestic helper)	
Play with children	Age, employment status, non-resident extended family size, hours child spends outside the home
Tutor children	As above
Number of housework activities	Age, employment status, non-resident extended family size, hours child spends outside the home, household income
Employment status by Domestic activities performed by non-residents / extended family interaction	As main effect models of domestic activities performed by non-residents / extended family
Domestic activities performed by spouse	
Play with children	Age, household composition, employment status, education, non-resident extended family size, hours child spends outside home
Tutor children	Age, household composition, employment status, education, non-resident extended family size, hours child spends outside home, non-residents/extended family: play with children
Number of housework activities	Age, household composition, employment status, education, non-resident extended family size, hours child spends outside home, non-residents/extended family: play with children, domestic helper
Employment status by Domestic activities performed by spouse interaction	As main effect models of domestic activities performed by spouse
Domestic activities performed by mother	

Play with children	Age, household composition, employment status, education, non-resident extended family size, hours child spends outside home, non-residents/extended family: play with children, residents: play with children, residents: housework activities, domestic helper, spouse: play with children; spouse: tutor children; spouse: housework activities
Tutor children	Age, household composition, employment status, education, non-resident extended family size, hours child spends outside home, non-residents/extended family: play with children, domestic helper, non-residents/extended family: tutor children; spouse: tutor children; residents: tutor children
Number of housework activities	Age, household composition, employment status, education, non-resident extended family size, hours child spends outside home, household income, domestic helper, non-residents/extended family: tutor children, spouse: tutor children, spouse: housework activities, residents: housework activities, residents: tutor children
Employment status by Domestic activities performed by mother interaction	As main effect models of domestic activities performed by mother

*covariates determined using directed acyclic graphs (see Additional file 1, Figure S1)
